# Multidimensional Stratification of *Severe Disability*: Demographic, Clinical, Geographic, Socio-Economic Profiles and Healthcare Pathways in a Cross-Sectional Italian Cohort

**DOI:** 10.3390/healthcare13243200

**Published:** 2025-12-07

**Authors:** Rita Chiaramonte, Tamara Civello, Giuseppe Laganga Senzio, Liberato Longo, Alessandro Santo De Caro, Fabrizio Li Gotti, Michele Vecchio

**Affiliations:** 1 Department of Biomedical and Biotechnological Sciences, University of Catania, 95123 Catania, Italy; 2 Azienda Sanitaria Provinciale (ASP) of Catania, 95100 Catania, Italy; 3 Physical Medicine and Rehabilitation Unit, AOU Policlinico G. Rodolico, University of Catania, 95123 Catania, Italy

**Keywords:** rehabilitation, disability, autonomy, equity, healthcare organization, sustainability

## Abstract

**Highlights:**

**What are the main findings?**

- This is the first study to systematically describe the stratification and management of individuals with severe disability in Sicily under the D.M. of 26 September 2016, using data from ASP Catania, in Sicily (Italy).
- The analysis integrates multiple dimensions—age, sex, healthcare district, type of disorders, and income level—revealing significant demographic, clinical, and territorial heterogeneity in the distribution and needs of the population.

**What are the implications of the main findings?**

- The findings highlight the need for integrated, personalized care models that are sensitive to age, gender, geographic, and socioeconomic differences, particularly in underserved districts.
- Addressing the complex needs of individuals with severe disability requires coordinated, multisectoral strategies that ensure equitable access to care while safeguarding social rights and economic sustainability.

**Abstract:**

**Background**: Individuals with severe disability require intensive and long-term healthcare, rehabilitation and social support. Updated population data are essential to inform planning and resource allocation. This study aimed to quantify—with a cross-sectional analysis conducted in 2025—the demographic, clinical, socioeconomic, and geographic characteristics of individuals with severe disability within the Provincial Health Authority (ASP) of Catania (Sicily) in Italy, and to identify statistically significant differences across subgroups. **Methods**: A cross-sectional analysis was conducted on 3277 individuals officially certified as having severe disability under the Italian Ministerial Decree of 26 September 2016. Data were extracted from administrative records and stratified by age, sex, clinical classification, income level, and healthcare district. Associations were tested using chi-square statistics. **Results**: Participants had a mean age of 39.14 ± 28.64 years; Minors represented 33% of the disability cohort (vs. 19.4% minors in the general provincial population) with a mean age 10.28 ± 3.55. Adults accounted for 67% of the cohort (vs. 81% adults in the general population), with a mean age of 69.94 ± 24.61 years. Females constituted 43% of the sample (compared with 51% females in the provincial population), whereas males represented 57% (vs. 49% males in the general population). Most individuals (95.9% of the cohort) had an income level below €25,000/year. **Conclusions**: The study reveals substantial demographic, socioeconomic, and clinical heterogeneity among individuals with severe disability and highlights significant district-level disparities. Notably, minors appear markedly over-represented in the disability cohort compared with the general population, while females are under-represented, indicating potential age- and sex-related differences in disability burden, access to assessment, or underlying diagnostic patterns. These findings indicate the need for stratified, district-sensitive planning approaches, ensuring equitable access to services and optimizing allocation of healthcare and social resources.

## 1. Introduction

People living with severe and complex disabilities represent one of the most vulnerable segments of society. Globally, an estimated 1.3 billion individuals—approximately 16% of the world’s population—live with significant disability [1], and these numbers continue to rise each year [2].

Among elderly with multimorbidity, the prevalence of disability has been reported at around 34.9%, with higher rates observed in women, unmarried individuals, and long-term users of healthcare service [2]. Although disability is often associated with aging, its burden is also substantial in children and adolescents, with major implications for population health [3].

Between 1990 and 2019, the global rate of disability-adjusted life years decreased by 79.8%, years lived with disability by 6.2%, and years of life lost by 89.3% across all causes [3]. Despite these improvements, disability, often related to unequal access to healthcare and resources, remain major public health concerns [4]. Current challenges include barriers to healthcare access, end-of-life care needs, financial instability, ageism and elder abuse, inadequate built environments, climate- and disaster-related threats, and social isolation [5].

Therefore, the management of severe disabilities entails significant clinical and social implications [6]. Existing interventions aimed at promoting equitable access to healthcare for persons with disabilities often fail to align with their actual needs. Sources of funding and long-term sustainability are often unclear, and only a limited number of initiatives provide targeted approaches to address the specific challenges of individuals with disabilities [6], particularly through multidisciplinary clinical protocols, specific rehabilitation programs and patients stratification strategies [7,8].

In Italy, the recognition and management of *severe disability* are regulated by the Ministerial Decree (D.M.) of 26 September 2016 [9], which specifies clinical criteria and validated assessment scales to ensure objective and standardized evaluation nationwide. In Sicily, regional health authorities apply these criteria to identify beneficiaries eligible for financial support and personalized care programs.

Despite the growing global attention to disability and long-term care needs, there is limited evidence on how populations with severe disability are distributed and managed within local health systems, particularly in Italy, where organizational models vary substantially across regions [10,11]. This study contributes new knowledge by providing the first multidimensional stratification of individuals with severe disability within a large Italian province, integrating epidemiological, socioeconomic, and organizational data. By examining clinical severity classifications, income-based eligibility, geographical distribution, and service assignment patterns, the study offers an evidence-based characterization of the population currently accessing *severe-disability* support. These findings fill a critical gap by identifying territorial and demographic variations that may inform resource allocation, equity assessments, and the design of integrated care models.

Given these challenges, this study aims to (1) describe the organizational framework and resource-allocation strategies implemented in the Sicilian Region for the management of individuals with severe disability, and (2) systematically stratify the population with *severe disability* within the Provincial Health Authority (ASP) of Catania. Specifically, the study assesses and compares demographic (age, sex), geographic (health district), socioeconomic (ISEE income brackets), and clinical variables (Ministerial Decree classification) to identify patterns of distribution and population stratification across districts and subgroups. 

## 2. Materials and Methods

This research was carried out in full compliance with the ethical standards outlined in the Declaration of Helsinki and received approval from the Local Ethics Committee “Catania 2” (protocol no. 92/CEL). It forms part of the PROMIS initiative, which seeks to advance best practices in clinical care and disseminate them [12]. The project is also integrated within the broader activities of the research networks of the ASP of Catania, *Smart Digitalization–OSLO System*, and *Internationalization Empowerment–Digit-Aid*, which contribute to the digital transformation and collaborative knowledge-sharing that underpin this work.

The study focused on individuals recognized as having *severe disability* under the criteria established by the M.D. of 26 September 2016 [9]. These individuals had applied for financial support and resided within the area served by the ASP of Catania, the second largest province in Sicily. The classification of individuals with very high care needs was based on validated clinical scales and standardized diagnostic criteria (Table 1).

This research is based on a cross-sectional analysis updated to December 2024, examining the distribution of cases and variations across different population categories and healthcare districts. The dataset included: (a) demographic variables such as age and sex, (b) clinical condition that led to the recognition of *severe disability* (Table 1), (c) Equivalent Economic Situation Indicator (income level ISEE), an Italian indicator used to assess household income and eligibility for social benefits, (d) healthcare district of residence corresponding to the administrative subdivisions of the ASP, which organize and structure the delivery of services within the regional health system. This study also captured participation in rehabilitation services, including home-based integrated care (*Assistenza Domiciliare Integrata*, ADI); multidisciplinary programs delivered in accredited rehabilitation centres (CdR) pursuant to Italian Law Article 26/1978 [13], offered in outpatient, semi-residential, or residential formats; and Residential Sanitary Assistance (RSA), which provides short-to medium-term residential healthcare, typically lasting one to two months.

The data of participants were included if they met the official criteria for *severe disability* as defined by the Italian Ministerial Decree of 26 September 2016 [9], which establishes specific clinical thresholds using validated assessment scales (e.g., Glasgow Coma Scale ≤ 10; Clinical Dementia Rating Scale ≥ 4; ASIA Impairment Scale A–B for cervical spinal cord injury; Expanded Disability Status Scale ≥ 9; Hoehn and Yahr stage 5; DSM-5 level 3 autism; IQ ≤ 34 or LAPMER ≤ 8). Only individuals formally recognized by the regional evaluation units and eligible for the corresponding financial benefit were considered.

Exclusion criteria were: (a) failure to meet the clinical thresholds established by the Ministerial Decree [9] and absence of formal eligibility for the regional severe-disability allowance; and (b) incomplete or inconsistent administrative records preventing accurate demographic, socioeconomic, or clinical stratification.

The analysis included demographic, socioeconomic, clinical, and healthcare-service–related variables. Demographic variables included age (measured in years and analyzed as continuous and categorical) and sex (female/male). Socioeconomic status was measured using the *Equivalent Economic Situation Indicator* (ISEE), an official Italian income-based index incorporating household income, assets, and composition. ISEE was categorized as <€25,000 (full eligibility for financial support) and ≥€25,000 (partial 30% reduction), in accordance with regional regulations. Geographic variables corresponded to the nine healthcare districts within ASP Catania (Acireale, Adrano, Bronte, Caltagirone, Catania, Giarre, Gravina, Palagonia, Paternò), which are the official administrative units responsible for service provision and resource allocation.

Clinical severity and diagnostic classification were operationalized through the categories defined in the Ministerial Decree of 26 September 2016 (Letters A–I, that reflects standardized, scale-based clinical judgement) [9]. These categories derive from validated clinical scales routinely applied by the regional Multidisciplinary Evaluation Units (UVM), including: Glasgow Coma Scale (GCS) [14] for disorders of consciousness, Clinical Dementia Rating Scale (CDRS) [15] for cognitive impairment, ASIA Impairment Scale for spinal cord injury [16], Expanded Disability Status Scale (EDSS) [17] for multiple sclerosis, Hoehn and Yahr scale (Hoehn & Yahr) [18] for Parkinson’s disease, DSM-5 severity levels [19] for autism spectrum disorder, IQ and LAPMER [20] for intellectual disability.

Healthcare-service variables included participation in: *Home Integrated Care (ADI)*, *Residential Healthcare Facilities (RSA)*, *Rehabilitation centres under Article 26/1978 (CdR)*—categorized by outpatient, semi-residential, residential, or home-based programs. Data were collected from records of individuals with *severe disability*, organized into a Microsoft Excel database, and analyzed using *R* statistical software (R version 4.5.). Descriptive statistics were applied to characterize the population across districts, age groups, sex, socioeconomic level, and clinical classification. Continuous variables (e.g., age) were summarized as mean ± standard deviation after verifying distributional characteristics. Categorical variables (e.g., sex, district, clinical categories, income groups) were reported as absolute frequencies and percentages. Associations between categorical variables were assessed using the chi-square test; when assumptions were not met, categories were not collapsed because of the epidemiological nature of the classifications, but results were interpreted with caution. Effect size for chi-square associations was expressed using the contingency coefficient. A two-tailed *p* < 0.05 was considered statistically significant. To improve comparability across demographic and geographic groups, disability frequencies were also expressed as relative proportions of the corresponding reference populations. This additional normalization allowed each subgroup to be interpreted in relation to its total population, providing contextualized prevalence estimates and enabling meaningful cross-district and cross-demographic comparisons.

**Table 1:** Clinical criteria and scales used to define very high care needs (D.M. 26 September 2016).

Disability Classification and Clinical Criteria (Ministerial Decree 26 September 2016) [9]	Clinical Distribution	N. of Adults	Mean Age Adults	N. of Underage	Mean Age Minors
A: Consciousness: Glasgow coma scale ≤ 10 [14]	9	9	52.88 ± 19.67	-	-
B: Ventilation: Dependence on invasive/non-invasive mechanical ventilation (24 h/7 days)	22	14	61.92 ± 18.83	8	5 ± 2.39
C: Dementia: Clinical Dementia Rating Scale ≥ 4 (severe/very severe) [15]	503	495	81.87 ± 11.25	8	8.75 ± 1.98
D: Spinal cord injury: Cervical spinal cord lesions between vertebrae C0 and C5, American Spinal Injury Association ASIA Impairment Scale A–B [16]	19	19	52.84 ± 15.02	-	-
E: Neuromuscular diseases: amyotrophic lateral sclerosis, advanced multiple sclerosis (Expanded Disability Status Scale score EDSS ≥ 9) [17], Parkinson’s stage 5 (Hoehn & Yahr) [18]	514	471	55.0 ± 22.02	43	11.58 ± 4.21
F: Sensory deprivation: Profound visual and auditory impairment	23	20	71.1 ± 18.47	3	13.66 ± 3.05
G: Neurodevelopmental disorders: Severe autism spectrum disorder (Diagnostic and Statistical Manual of Mental Disorders DSM-5, level 3) [19]	893	214	27.51 ± 10.74	679	9.95 ± 3.20
H: Profound intellectual disability (Intelligence quotient IQ ≤ 34; Lapmer-Level of Activity in Profound/Severe Mental Retardation LAPMER ≤ 8) [20]	1096	810	40.24 ± 15.79	286	11.30 ± 3.79
I: Any condition of vital dependency requiring permanent care and monitoring	198	136	61.66 ± 23.79	62	8.90 ± 4.03

## 3. Results

### 3.1. Regulatory Framework, Organizational Models, and Resource Allocation Strategies

In Sicily, resource allocation relies on regional funds for non-self-sufficient individuals. Economic support is provided through monthly allowances and personalized care plans. Core services include financial allowances to compensate family members or caregivers for continuous assistance; ADI and RSA, which provide nursing, rehabilitation, and social support at home or in residential settings, respectively; CdR pursuant to Italian Law 26/1978 [13], offering rehabilitation in outpatient, semi-residential, or residential regimens; and the provision of medical devices (e.g., ventilators, feeding systems, assistive technologies) as established by the national Essential Levels of Care (LEA), which guarantee specific health services and assistive technologies to all citizens with disabilities through the National Health Service (Italian Law 833/1978 and subsequent amendments) [13].

The access to economic support for individuals with very high care needs is regulated through an integrated framework that combines healthcare and social services. Information on service organization and financial management was obtained from official ASP guidelines, regional regulations, and publicly available administrative circulars, with specific reference to M.D. 26 September 2016 [9], the Presidential Decree of the Sicilian Region No. 589/2018 [21] and the Interdepartmental Circular No. 5/2021 issued jointly by the Regional Health Department and the Department of Family and Social Policies [22].

Applications can be submitted by patients and caregivers twice a year (first semester: January–June; second semester: July–December) to the local Health District. Then, each request is evaluated by the Multidimensional Assessment Unit (UVM) of the health districts, which verifies whether the applicant meets the criteria of “very high care needs” as defined in Article 3 of D.M. 26 September 2016 [9]. Deadlines for completing UVM assessments and clinical evaluations are set within three months of submission: September 30 for first-semester applications and March 31 of the following year for second-semester applications. If eligibility is confirmed, beneficiaries (or their legal representatives) must sign a care agreement before the allowance is granted. This system is designed to ensure that support is both needs-based and periodically reassessed, reflecting the complexity and dynamic nature of care for individuals with *severe disability* (Table 2).

### 3.2. Stratification of Severe Disability Within ASP Catania

In accordance with the predefined objectives and variable definitions reported in the Methods, the following section presents the descriptive stratification of the full population of individuals with severe disability within the ASP of Catania. Results are organized by demographic (age, sex), geographical (district of residence), socioeconomic (ISEE income levels), and clinical variables (diagnostic classification according to the Ministerial Decree). All analyses directly reflect the measurement and comparative groups defined in the methodology framework.

As of September 2025, 3277 individuals with *severe disability* were receiving economic support. Adults accounted for two-thirds of the population with disability (2188; 66.8% vs. 80.6% of the adult population in the province of Catania) with a mean age of 53.50 ± 24.51 years, while minors represented one-third (1089; 33.2% of the disability cohort (vs. 19.4% minors in the general provincial population) with a mean age of 10.28 ± 3.55 years. Women represented 43.3% of individuals with severe disability (vs. 51.4% females in the provincial population) and 56.7% males (vs. 48.6% males in the general population) (Table 3).

As showed in Table 4, the comparison between district-level demographic structures and the age distribution of individuals with *severe disability* reveals meaningful and policy-relevant disparities. The systematic over-representation of minors in the severe-disability population—often more than double their proportion in the general district population—suggests that severe disability in this cohort is strongly driven by early-onset, lifelong conditions such as intellectual disability, autism spectrum disorders, and complex neurodevelopmental syndromes. Conversely, adults are relatively under-represented compared with expected demographic baselines, which may reflect differences in survival, migration, or service-access mechanisms. This imbalance highlights the need to investigate potential service gaps in adult disability care, including long-term rehabilitation, residential support, and integrated socio-health pathways.

**Figure 1:**
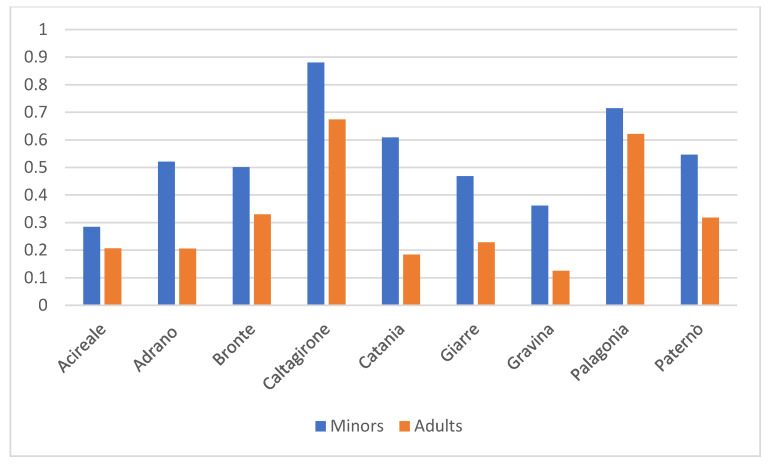
Relative Age Distribution Across Healthcare Districts in the ASP of Catania Based on Total Population.

District distribution reflected underlying population size. The largest numbers were found in Catania (533 adults out of 289,446 of the adult population of the district, 427 minors of 70,184 of total minors of the districts), followed by Caltagirone (411 adults of 61,028 of the total adult population, 117 minors of 132,88 of total minors of the district) and Palagonia (278 adults out of 44,755 total adults of the district, 78 minors of 10,915 total district minors). Smaller districts, such as Bronte (96 adults of 29,116, 33 minors of 6587) and Adrano (103 adults of 50,105, 74 minors of 14,201), showed a relatively higher proportion of minors (Figure 1).

**Figure 2:**
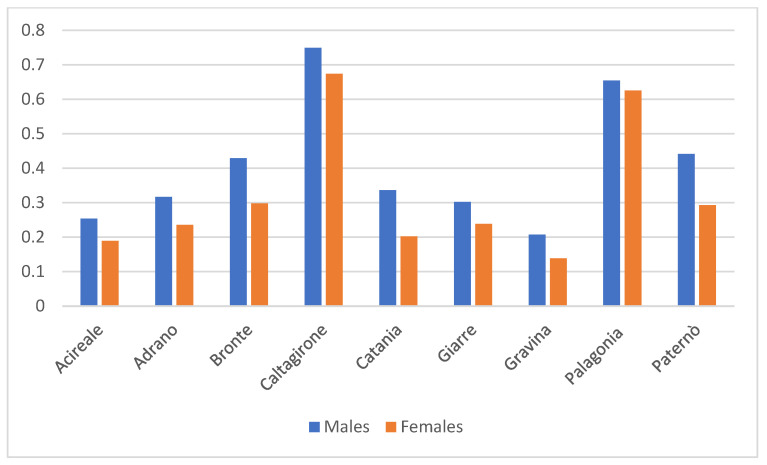
Relative Gender Distribution Across Healthcare Districts in the ASP of Catania Based on Total Population.

Males were consistently more represented than females in the disability population across all districts (overall: 56.7% males vs. 43.3% females), a pattern that contrasts with the general population of the province, where females are slightly more numerous (48.6% males vs. 51.4% females). When examining district-level patterns, the relative proportion of males with severe disability exceeded that of females in every district, ranging from 52–61% males and 39–48% females (Acireale 56/44%, Adrano 56/44%, Bronte 57/43%, Caltagirone 52/48%, Catania 61/39%, Giarre 55/45%, Gravina 58/42%, Palagonia 51/49%, Paternò 59/41%). Notably, this male predominance persists even in districts where the general population shows a more balanced sex distribution or a slight female majority. The comparison between absolute population data and disability-specific proportions therefore confirms that the male excess is not a reflection of district demographic structure but represents a true epidemiological over-representation of males within the severe-disability cohort (Figure 2).

Most individuals (3143, 95.9% of people with *severe disability*) had an ISEE below €25,000/year, only 134 individuals (4.1% of people with *severe disability*) had an ISEE ≥ €25,000/year. Districts with the largest numbers in the lower bracket included Catania (922 with ISEE < €25.000; 38 with ISEE ≥ €25.000), and Caltagirone (511 and 17, respectively), followed by Palagonia (346 and 10, respectively). Smaller clusters were observed in Bronte (121 with ISEE < €25.000 and 8 with ISEE ≥ €25.000) and Adrano (173 and 4). Among females, 1346 (94.8% of people with *severe disability*) were in the lower ISEE group and 74 (5.2% of disable people) in the higher, whereas among males 1797 (96.8% of disable people) were in the lower group and 60 (3.2% of disable people) in the higher. Nevertheless, the overwhelming majority of both sexes belonged to the lower ISEE group, underscoring that this remains a socioeconomically fragile population. These distributions confirm a marked socioeconomic vulnerability in this population.

The distribution of patients across the diagnostic categories defined by the M.D. of 26 September 2016 [9] and health districts reveals meaningful patterns. The two most prevalent categories—H, referring to profound intellectual disability (1.096 disable people; 33.4% of people with *severe disability*), and G, related to neurodevelopmental disorders and autism spectrum disorder (893 disable people; 27.3% of people with *severe disability*), were consistently the most frequent across districts, particularly in Catania (H: 342; G: 353) and Caltagirone (H: 141; G: 100). Category E accounted for 514 patients (15.7%), with substantial representation in Catania and Caltagirone, whereas category C included 503 patients (15.3%) affected by amyotrophic lateral sclerosis and advanced multiple sclerosis Parkinson’s disease. Less common categories included I (198 individuals with devices of vital dependency; 6.0% of disable people), D (19 with cervical spinal cord injury), A (9 with consciousness disorders; 0.3%), B (22 in mechanical ventilation; 0.7%), and F (23 with sensory deprivation; 0.7%). Smaller districts such as Adrano, Bronte, and Acireale mirrored this pattern, with a preponderance of patients in categories H, G, E, and C, and sparse representation in the rarer categories. Indeed, sex distribution varied across categories: females were more represented in categories C (70.2% females, 29.8% males) and E (50.2% females, 49.8% males), whereas males predominated in G (77.2% males, 22.8% females) and D (68.4% males, 31.6% females). In H, the largest subgroup (33.4% of the people with *severe disabilities*), the distribution was more balanced but still male-skewed (56.2% males, 43.8% females). Smaller groups such as A, B, and F were numerically limited but also showed slight male predominance.

Regarding care settings, CdR delivered ambulatory services to 336 minors and 64 adults, home-based services to 285 adults and 56 minors, and semi-residential care to 50 minors and 266 adults. One adult was under evaluation for potential full residential placement. In addition, 287 adults and 31 minors were managed through the ADI, while 9 adults received care in RSA.

## 4. Discussion

To the best of our knowledge, this is the first study to systematically describe the stratification and management of individuals with *severe disability* in Sicily related to the D.M. of 26 September 2016. This study provides a cross-sectional overview of individuals with *severe disability* within the ASP of Catania, one of Sicily’s largest provinces, offering insights that may reflect broader regional and national trends. These findings must be interpreted within the analytical boundaries of the study: the observed geographical and demographic differences reflect the distribution of the variables as collected in administrative records and do not imply causal relationships. Instead, they highlight measurable disparities in the composition of the population with severe disability across districts, directly addressing one of the core study objectives.

The age stratification reveals a predominance of adult cases, consistent with the cumulative impact of chronic and degenerative conditions in aging populations [2]. However, in peripheral districts such as Adrano and Bronte, minors represent a larger proportion, indicating localized needs for paediatric rehabilitation and tailored support services. These findings emphasize the importance of age-sensitive care models that address both adult and paediatric populations. Indeed, proactively addressing the public health challenges posed by rapidly ageing populations worldwide requires collaborative, multisectoral policy solutions that promote healthy, equitable, and socially engaged ageing. Healthcare systems, communities, and policies must be optimized both to meet the needs of older adults and to leverage their strengths [5]. The same principle applies to children, who equally require equitable, personalized, and socially inclusive care pathways [3].

The comparison between absolute frequencies and population-adjusted indicators provides important insights into the demographic structure of individuals with severe disability in the province of Catania. Although adults constitute two-thirds of the disability cohort in absolute terms, the relative analysis shows that minors are substantially over-represented when compared with the demographic composition of the general population. This discrepancy suggests that severe disability in this region is predominantly driven by early-onset, lifelong conditions, which become clinically evident during childhood and require continuous assistance across the life course. Conversely, adults appear under-represented, which may reflect differences in diagnostic pathways, survival rates, or access to care. These findings underscore the necessity of interpreting absolute numbers with caution, as they may obscure important epidemiological patterns that emerge only once relative proportions are considered. Similarly, the sex distribution highlights an important divergence between the disability population and the general population. The male over-representation is consistent with the higher prevalence of certain early-onset neurodevelopmental conditions—such as autism spectrum disorder and profound intellectual disability—which disproportionately affect males. These differences not only align with known epidemiological trends but also further support the observation that the disability population is shaped by conditions emerging in childhood, thereby reinforcing the relevance of age-adjusted and gender-adjusted analyses in interpreting service needs.

Disorders distribution shows a high prevalence of severe neurological impairment and high dependency, reflecting the growing burden of chronic diseases, multimorbidity, and disability, alongside increasing rates of dependency, mental health disorders, caregiving shortages, and deficiencies in long-term care systems [5]. Intellectual disability (category H) and neurodevelopmental disorders (G) are especially concentrated in urban centres like Catania and Caltagirone, possibly due to better service availability or underreporting in less-resourced areas [23]. Gender differences also emerged, with women more frequently affected by dementia and neuromuscular diseases, as multiple sclerosis, Parkinson disease and amyotrophic lateral sclerosis (C, E), while men showed higher rates of motor and cognitive impairments (G, D, H), including spinal cord injuries and intellectual disability, more frequent conditions among men [24].

Socioeconomic analysis confirms that most individuals with *severe disability* belong to low-income households, reinforcing the association between disability and economic vulnerability [25]. Broader health inequities—such as disparities in access, mortality, and social determinants—further underscore the urgency of equitable service provision [4]. Moreover, the rising trends in chronic disease and related healthcare expenditures in Italy, as well as in other developed countries, highlight the urgent need for coordinated clinical and social interventions [26,27].

Despite Italy’s regulatory framework promoting integration between health and social services, regional disparities persist. Southern regions, including Sicily, continue to report higher disability-adjusted life years and premature mortality, as years of life lost, compared to the north, driven by socioeconomic deprivation and weaker health infrastructure [28]. Contributing factors include socioeconomic deprivation, weaker health infrastructure, and lower healthcare investment—all of which adversely impact the equitable delivery of disability support services [11].

International comparisons reveal similar challenges: in Australia, delays in accessing home care packages often lead to premature institutionalization and increased mortality risk [29,30]. In Italy, efforts to strengthen integrated care have advanced through services such as RSA, ADI and CdR, increasingly supported by digital technologies and innovative home care strategies [31,32].

This pattern has important implications: districts with a disability profile require long-term, continuity-of-care planning, expanded specific and transitional services, and sustained investment in rehabilitative, and family-support networks. From a health-system perspective, the alignment—and often misalignment—between the demographic structure of the general population and the composition of the disability cohort provides essential contextual information for resource allocation and planning. These results demonstrate that district-level strategies should not rely solely on absolute disability counts; rather, they must incorporate population-adjusted indicators to design equitable, efficient, and age-sensitive services tailored to the actual needs of local communities. Overall, the findings underscore the need for coordinated, multisectoral approaches that respond to the complex needs of individuals with *severe disability*, while highlighting how existing disparities make it imperative to carefully balance financial sustainability with the protection of social rights—ensuring that economic thresholds do not translate into inequities in access to essential care and support [33].

The present analysis carries inherent limitations that should be considered when interpreting the findings. First, the analysis was limited to a single province and, although Catania is the second largest Sicilian province, the findings may not fully capture broader trends; however, the sample represents a population that can be reproducibly scaled. Second, reliance on administrative data may introduce classification or reporting biases, as diagnostic and functional assessments depend on the accuracy, fidelity, and reliability of physician evaluations and registries. Third, although relative frequencies were calculated for demographic and geographic variables, it was not possible to produce comparable relative estimates for socioeconomic status, as general population-level ISEE distributions are not available for the province. Consequently, interpretations regarding economic vulnerability are limited to the disability cohort and cannot be contextualized against the general population. Finally, these socioeconomic indicators were limited to the income level ISEE income level, which, although standardized in Italy, may not fully represent household wealth, informal caregiving, or access to non-documented resources.

## 5. Conclusions

This study provides a comprehensive portrait of individuals with *severe disability* within the ASP of Catania, highlighting their demographic distribution, clinical profiles, and patterns of rehabilitation service utilization within the regional framework. Although conducted in a single province, the findings reflect broader dynamics that intertwine medical complexity with socio-economic vulnerability.

The observed associations between sex, age, income level, and disability classification underscore the heterogeneity of this population and the urgent need for personalized, integrated care strategies. Sustained investment and long-term planning must be sensitive not only to clinical severity but also to demographic and social determinants, ensuring equity in access and efficiency in resource allocation. Persistent local challenges mirror global patterns, particularly as multimorbidity rises in ageing populations. This calls for healthcare systems to evolve, embracing long-term planning, sustained investment, and innovative models of care that balance financial sustainability with the protection of social rights and human dignity.

Future comparative studies across regions would be instrumental in identifying best practices and supporting the development of integrated, equitable, and sustainable care models for individuals with *severe disability*.

## Figures and Tables

**Table 2:** Administrative pathway for financial support at ASP Catania.

Step	Applicants	Responsible, Evaluators	Timeline	Periods
1. Applications	Patients and caregivers	ASP Catania–Disability Unity, Department of Rehabilitation	Twice per year	January–June (1st semester); July–December (2nd semester)
2. Clinical Assessment	Patients	Specialist physicians of the UVM within ASP health districts	Within 3 months after submission	By September 30 from application (for 1st semester); by March 31 of the following year (for 2nd semester)
3. Care Agreement	Beneficiaries or legal representatives	Healthcare Districts of ASP Catania	Upon approval of eligibility	Mandatory for activation of the economic benefit
4. Payment	Patients	ASP CT-Sicily Region	Following signing of the Care Agreement	From October 1 (for 1st semester); from April 1 of the following year (for 2nd semester)
5. Check	-	ASP Catania–Disability Unity, Department of Rehabilitation	Continuous–verification of ISEE (<€25,000: full benefit; ≥€25,000: 30% reduction), incompatibility with other INPS allowances, and suspension during hospitalization	Ongoing

Multidimensional Assessment Unit UVM; Provincial Health Authority ASP; Equivalent Economic Situation Indicator ISEE.

**Table 3:** Demographic, clinical characteristics, and organizational and economic management strategies.

**Demographic Characteristics**		**Organizational and Economic Management**	
		**ISEE ≥ 25.000 €**	N. of *Severe Disability* People 134
**Adults with Disability** **Adults of Total Population**	67%; 69.94 ± 24.61 y 81%	**ISEE < 25.000 €**	N. of *Severe Disability* People 3143
**Minors** **Minors of** **Total Population**	33%; 10.27 ± 3.61 y 19%	**Health Districts**	**N. of Severe Disability People**
**Females** **with Disability/Total Female Population**	43%/51%	Acireale	299
**Males** **with Disability/Total Male Population**	57%/49%	Adrano	177
**Care Setting**	**Minors–Adults**	Bronte	129
CdR Ambulatory Outpatients	336–64	Caltagirone	528
CdR home care	56–285	Catania	960
CdR semi-residential care	50–266	Giarre	225
CdR full residential care	0–1	Gravina	320
ADI	31–287	Palagonia	356
RSA	0–9	Paternò	283

Equivalent Economic Situation Indicator ISEE; Rehabilitation centres CdR; Home-based integrated care programs ADI; Residential sanitary assistance RSA; Relative percentage-of disable people-rel. (%); Absolute percentage-of the total population in the Catania Province-abs. %; Number N; Years Old y.

**Table 4:** Distribution of Severe Disability by Age Group Across Healthcare Districts of the Catania Province.

Healthcare Districts	Relative % of Disables				Absolute Number			
	Minors	Adults	Males	Females	Minors	Adults	Males	Females
Acireale	24%	76%	56%	44%	18%	82%	56%	44%
Adrano	42%	58%	56%	44%	22%	78%	56%	44%
Bronte	26%	74%	57%	43%	18%	82%	57%	443%
Caltagirone	22%	7 8%	52%	48%	18%	82%	52%	48%
Catania	44%	56%	61%	39%	20%	80%	61%	39%
Giarre	30%	70%	55%	45%	17%	83%	55%	45%
Gravina	42%	58%	58%	42%	20%	80%	58%	42%
Palagonia	22%	78%	51%	49%	20%	80%	51%	49%
Paternò	31%	69%	59%	41%	21%	79%	59%	41%

## Data Availability

The data used in this study are confidentially available, although in aggregate and anonymous form, upon motivated request. Public sharing of data is not permitted due to institutional regulations on the use of health databases.

## Abbreviations

The following abbreviations are used in this manuscript:

ASP	Provincial Health Authority
D.M.	Ministerial Decree
ADI	Home-based integrated care
CdR	Accredited rehabilitation centres
LEA	Essential Levels of Care
UVM	Multidimensional Assessment Unit
RSA	Residential Sanitary Assistance
